# Supplementary material for: Cloud BioLinux: pre-configured and on-demand bioinformatics computing for the genomics community
Source: BMC Bioinformatics. 2012 Mar 19;13:42. doi: 10.1186/1471-2105-13-42 (PMC3372431; doi:10.1186/1471-2105-13-42)
Supplement: Additional file 1 — Supplementary 1 Cloud BioLinux software documentation in the form of a mini, self-contained website. Users need to download and uncompress the .zip file, and open through a web browser the "index.html" file available on the main directory. (ZIP 1823 kb). [file 1471-2105-13-42-S1.ZIP › Cloud-BioLinux-Package-Documentation/docs/mrbayes-multi.html]

Bio-Linux Software Documentation Pages

Back to search form

## mrbayes-multi

|  |  |
| --- | --- |
| Name | mrbayes-multi |
| Description | **MrBayes** is a program for the Bayesian estimation of phylogeny. **mrbayes-multi** provides a multi-processor version of MrBayes.  In order to run the parallel version of Mr Bayes (across multiple processors) you must have mpi and installed, and MrBayes must be launched using the mpirun command (i.e. it cannot be executed in the standard interactive mode). Finally you will need to have mpd configured on your system.  To achieve the above, you can run commands similar to the following: `echo "MPD_SECRETWORD=secret"  ~/.mpd.conf  chmod 600 ~/.mpd.conf  mpd &  mpirun -np 4 mrbayes-multi`  In the first command above, you will want to change the text "secret" to a password only you know. In the final command, change the number 4 to the number of cores you wish to run mrbayes over.  Bayesian inference of phylogeny is based upon a quantity called the posterior probability distribution of trees, which is the probability of a tree conditioned on the observations. The conditioning is accomplished using Bayes's theorem. The posterior probability distribution of trees is impossible to calculate analytically; instead, MrBayes uses a simulation technique called Markov chain Monte Carlo (or MCMC) to approximate the posterior probabilities of trees. |
| Homepage |  |
| Remote Documentation | http://mrbayes.csit.fsu.edu/wiki/index.php/Main\_Page |
